# Anatomic 2-Incision Distal Biceps Technique Using Unicortical Buttons

**DOI:** 10.1016/j.eats.2024.103343

**Published:** 2024-12-27

**Authors:** Andrew Renshaw, Evans Few, Noah Schneider, Karim Meijer

**Affiliations:** From the Ochsner Andrews Sports Medicine Institute, New Orleans, Louisiana, U.S.A.

## Abstract

Most patients with distal biceps tendon ruptures would benefit from surgical intervention, however, there has yet to be a clear consensus regarding the optimal technique and method of distal biceps fixation. Techniques can be broadly classified by approach—1-incision or 2-incision approach—and method of fixation—including options such as bone tunnel, cortical button, suture anchor, and interference screw fixation. In this article, we describe a technique for anatomic fixation of the distal biceps tendon, utilizing 2 looped sutures and 2 unicortical suture buttons. The described technique is performed through a muscle-splitting, 2-incision approach but can be performed through a 1-incision approach.

Distal biceps tendon ruptures have been estimated to occur in approximately 1.2 per 100,000 people per year.^1^ These injuries are most common in the dominant arm of males between 40 and 50 years old.^1^^,^^2^ Because deficits are poorly tolerated in this patient population, surgical intervention is the standard of care for the treatment of distal biceps rupture, as numerous studies have shown superior functional outcomes in terms of patient-reported outcomes, improved strength, particularly with supination, and reduced fatigability at the elbow after surgical repair.^2^, ^3^, ^4^, ^5^, ^6^

One of the primary goals of surgery includes the restoration of anatomy. Cadaveric studies have shown that the distal biceps tendon is a broad, ribbon-shaped structure that inserts on the ulnar aspect of the radial tuberosity. It consists of the long head biceps tendon, which inserts proximally, and short head biceps tendon, which inserts distally; total footprint dimensions are approximately 7 mm in width and 20 mm in length.^7^ The relative positions of the long and short head insertions are important for function, as the long head inserts further away from the axis of forearm rotation, providing a fulcrum for supination, while the short head inserts distally, increasing the leverage for flexion across the elbow.^7^^,^^8^

While surgical intervention has shown improved outcomes for distal biceps rupture, there is no clear consensus regarding optimal surgical technique. Distal biceps tendon repair was originally described utilizing a 1-incision approach to the antecubital fossa,^9^ but the incidence of posterior interosseous neuropathy led to the development of a 2-incision technique.^10^ Proponents of the 2-incision approach for distal biceps repair note improved access to the anatomic footprint of the biceps tendon insertion and decreased rates of posterior interosseous nerve injury as key advantages of the 2-incision approach; however, some cases of heterotopic ossification and proximal radioulnar synostosis occurred with the use of this technique (Table 2).^11^ Subsequently, a muscle-splitting 2-incision approach was developed to avoid subperiosteal dissection from the ulna to radial tuberosity,^12^ and this modification led to a decrease in the incidence of heterotopic ossification and synostosis.^13^

**Table 2:** Advantages/Disadvantages

- This technique is performed through 2 small incisions and allows for anatomic reconstruction of the short and long heads of the distal biceps tendon. - The 2-incision technique has lower rates of lateral antebrachial cutaneous nerve injury. - The use of intramedullary buttons creates robust fixation and allows for an on-lay effect for tendon-to-bone healing. - Patients are allowed early range of motion and are generally cleared for a full return to activity at 4 mo postoperatively. - Although this technique has been utilized for chronic and revision cases, a more extensile proximal incision than that described may be required in cases with significant tendon retraction or atrophy. - Although no cases of radioulnar synostosis have occurred in the senior surgeon’s experience, the historical concern for heterotopic ossification and synostosis in the 2 incision technique remains. - There is increased cost with the use of 2 cortical buttons.

Additionally, several methods have been developed for fixation of the distal biceps tendon to the radial tuberosity. These include bone tunnel, cortical button, suture anchor, and interference screw fixation. A systematic review by Chavan et al.^14^ found that cortical button fixation had a higher load to failure and stiffness than bone tunnels, intraosseous screws, and suture anchors. Watson et al.^15^ found that bone tunnel and cortical button fixation have lower complication rates than suture anchor and intraosseous screw fixation. Forlenza et al.^16^ reported improved failure strength and stiffness with the use of 2 unicortical intramedullary buttons.

Herein, we present a technique for anatomic fixation of the distal biceps tendon, utilizing 2 looped sutures and 2 unicortical buttons to recreate the 2 heads of the biceps tendon insertion (Video 1). While this fixation method may be employed with a 1-incision or 2-incision approach, the senior author chooses to utilize a muscle-splitting, 2-incision approach, noting more reliable anatomic restoration of the footprint for each of the 2 heads of the biceps tendon, decreased lateral antebrachial cutaneous nerve complications, and the ability to treat both acute and chronic ruptures with this technique.

## Technique

### Patient Evaluation

Distal biceps tendon ruptures classically present with an acute “pop” or tearing sensation at the elbow with onset of pain, often accompanied by weakness. This is caused by an excessive eccentric contraction of the biceps muscle while the elbow is forcibly extended from a flexed position with forearm supination. Patients may develop ecchymoses over the antecubital fossa and a “reverse Popeye deformity,” characterized by proximal retraction of the biceps muscle belly. The hook test is a sensitive and specific physical exam maneuver for assessing distal biceps disruption.^17^ For partial thickness injuries or chronic pathology, ultrasound or magnetic resonance imaging may be utilized for further evaluation.

### Surgical Technique

**Fig 1:**
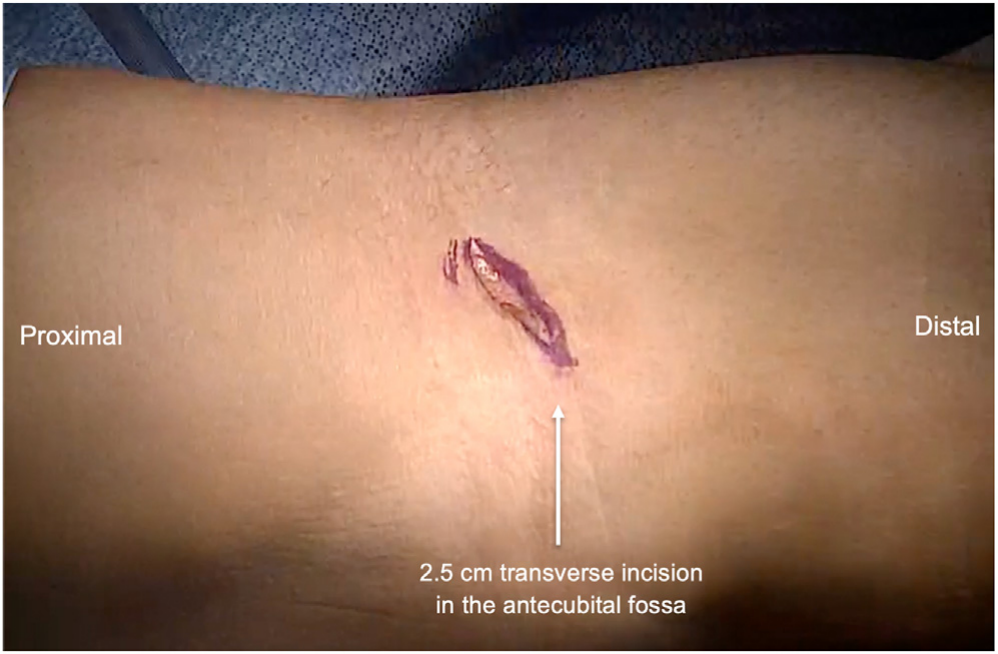
The patient is positioned supine with a hand table attachment. Once the operative extremity has been prepped and draped, a 2.5-cm transverse incision is made in the antecubital fossa with the elbow extended and supinated.

**Fig 2:**
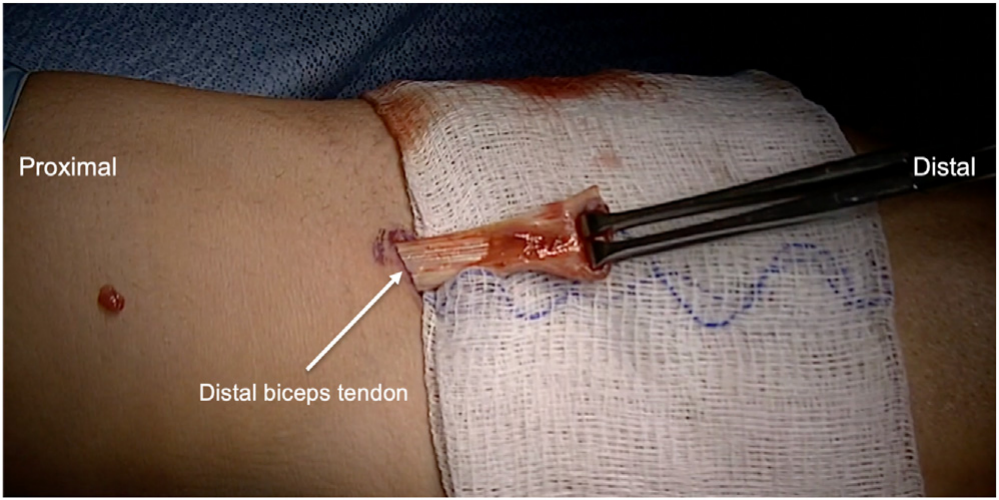
Viewing the volar aspect of the forearm with the elbow extended and supinated, the biceps tendon sheath is incised and the distal biceps tendon is identified. An Allis clamp is used to grasp the edge of the tendon and bring the tendon into the surgical field by pulling gentle longitudinal traction.

**Fig 3:**
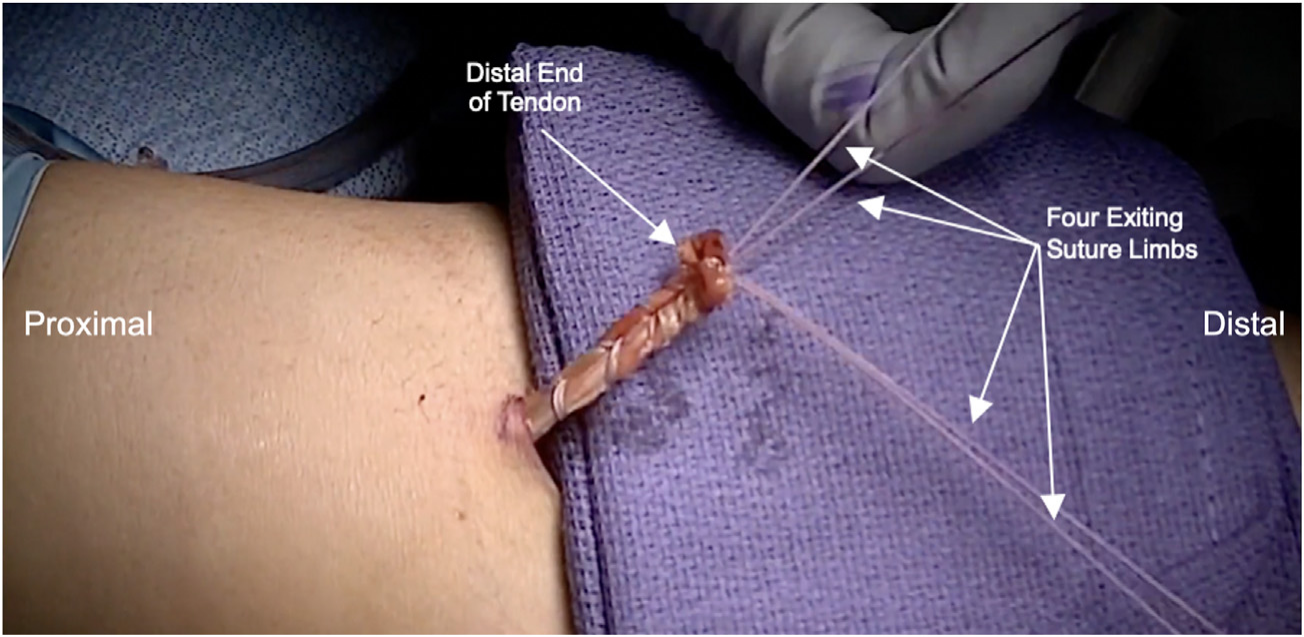
The distal biceps tendon is then sutured proximal to distal beginning just distal to the myotendinous junction via a standard looped baseball stitch. The free suture ends are then locked by using a free needle and the surgeon is left with 4 exiting suture limbs, 2 from each head of the distal biceps.

The patient is positioned supine on an operating room table or hospital stretcher with a hand table attachment. A nonsterile tourniquet may be used. A 2-cm transverse incision is made over the antecubital crease (Fig 1), and blunt dissection is used to gain exposure to the biceps tendon sheath, taking care to identify and protect important structures such as the lateral antebrachial cutaneous nerve and cephalic vein. The biceps tendon sheath is incised, and then the biceps tendon is grasped with an Allis clamp (Fig 2). The tendon may be debrided to remove diseased tendons and tenosynovitis. Then, the tendon is sutured using two No. 2 FiberLoop sutures on a straight needle (Table 1; Arthrex, Naples, FL). Starting just distal to the myotendinous junction of the biceps, the two No. 2 FiberLoop sutures needles are passed through the tendon in unison to create a standard looped baseball stitch. Five suture passes are made, proceeding distally. Then the looped end with the straight needle is cut from the 2 looped sutures, leaving 4 exiting limbs from the distal aspect of the distal biceps tendon (Fig 3). A free curved needle is used to pass each of the 4 suture limbs behind the last baseball throw, creating a locking stitch. Two of the suture limbs are used to recreate the short head biceps tendon, and the other 2 suture limbs are used to recreate the long head biceps tendon.

**Table 1:** Pearls/Pitfalls

- Required Equipment: 3.2-mm drill pin x2, 7-mm cortical button x2, #2 looped Fiberwire x2. (Arthrex, Naples, FL) - Release the lacertus fibrosus in cases of partial tear or chronic injury in order to mobilize the distal biceps tendon. - Use a Kelly clamp within the distal biceps tendon sheath to approach the radius. By ensuring that the approach is made directly to the radius, there is a decreased risk of violating the ulnar periosteum and causing a radioulnar synostosis. - Copiously irrigate the distal incision after drilling the radius in order to remove any bone debris and marrow elements from drilling, decreasing the risk for heterotopic bone formation. - As the Kelly clamp is retracted, it can be opened slightly to dilate the opening in the interosseous membrane, which will allow easier passage of the biceps tendon through the interosseous membrane. - If it is difficult to pass the tendon, the forearm may be gently pronated and supinated to aid in the passage of the biceps tendon through the interosseous membrane. - Once the distal biceps tendon is passed, gentle traction is applied to the tendon to ensure that there is adequate excursion of the tendon without adhesions.

**Fig 4:**
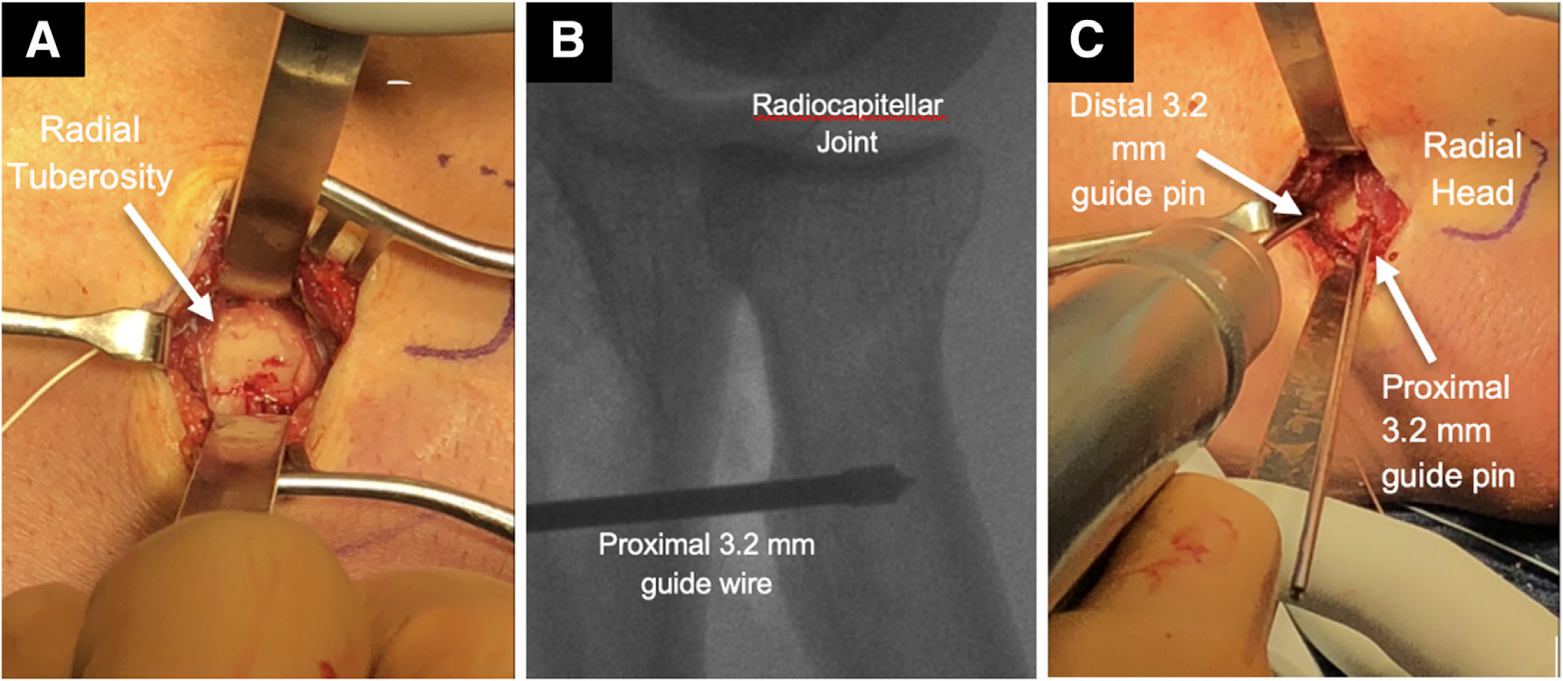
(A) Viewing the radial tuberosity from the dorsolateral aspect of the forearm with the elbow in full pronation, 2 Hohmann retractors are placed on the volar and dorsal side of the radial tuberosity to improve visualization and identify the footprint of the long and short head of the biceps. (B) Fluoroscopic image of proximal pin insertion to confirm correct positioning distal to the radiocapitellar joint but within the proximal aspect of the radial tuberosity. (C) Starting proximally, a 3.2-mm guide pin is placed into the radial tuberosity at the insertion of the long head of the biceps. A second 3.2-mm guide pin is placed distally at the insertion of the short head of the biceps.

**Fig 5:**
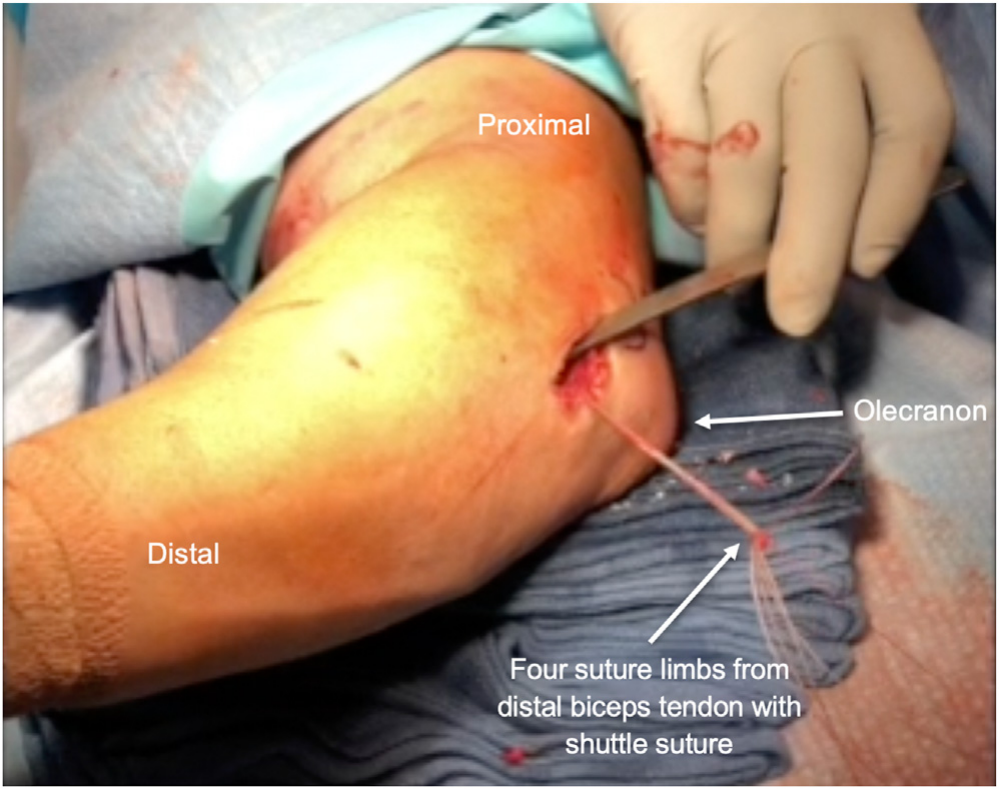
When viewing with the elbow in flexion and maximal pronation, the 4 suture limbs from the distal biceps tendon are pulled from volar to dorsal, through the interosseous membrane, and out of the incision at the dorsolateral aspect of the forearm overlying the radial tuberosity. A shuttle suture is utilized for the passage of the suture and tendon.

**Fig 6:**
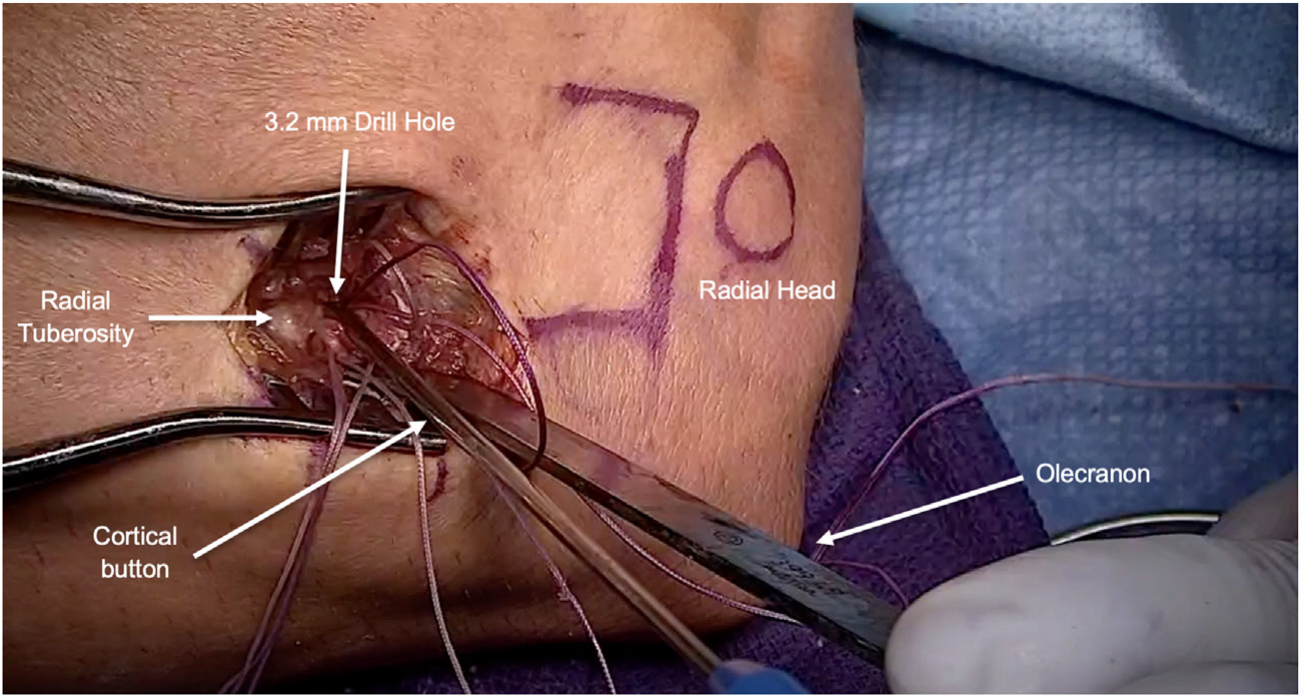
When viewing from the dorsolateral with the elbow in flexion and pronation, a cortical button is loaded onto each pair of exiting suture limbs (2 cortical buttons total). The cortical button is advanced through the previously created 3.2-mm drill hole in the radial tuberosity and flipped intramedullary. Each pair of suture limbs is then tensioned by hand.

**Fig 7:**
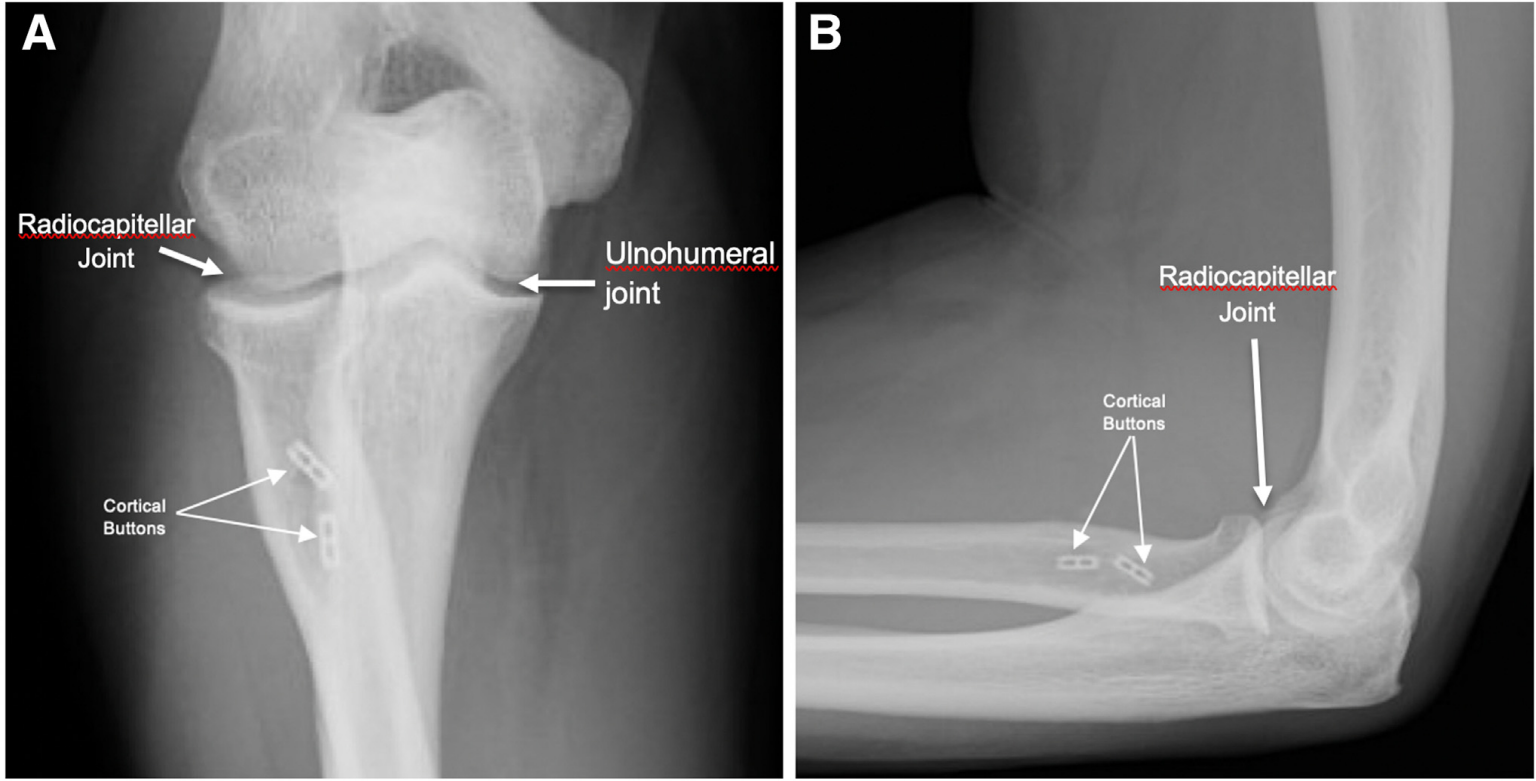
(A) AP radiograph of the elbow after tensioning of intramedullary suture buttons at the radial tuberosity. (B) Lateral radiograph of the elbow showing intact intramedullary suture buttons at the level of the radio tuberosity following final tightening.

**Fig 8:**
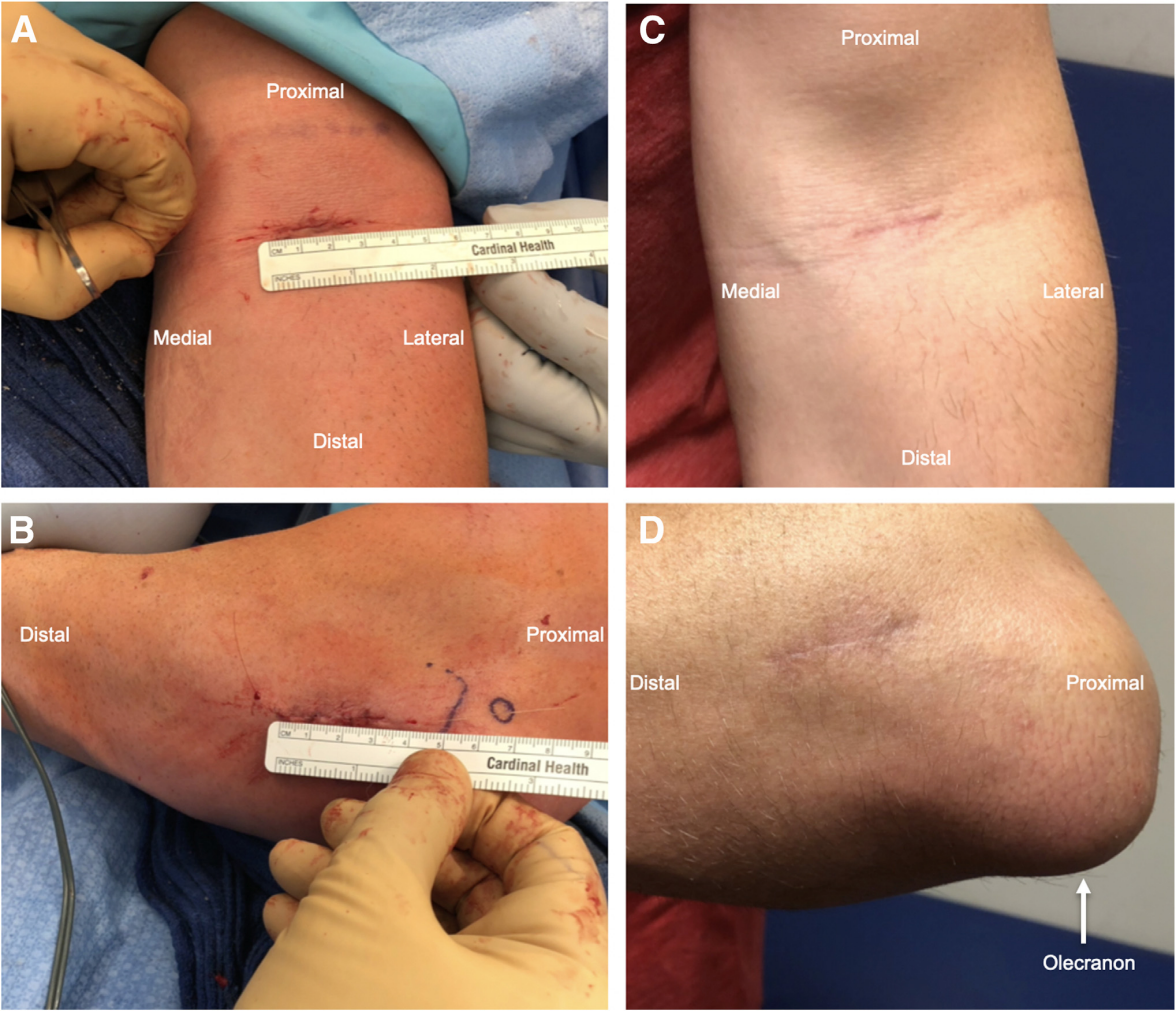
Postoperative closure of the 2.5-cm incision on the antecubital fossa (A) and 3-cm incision on the dorsolateral forearm (B). (C) Fully healed incisions of the antecubital fossa and (D) the dorsolateral forearm.

Next, attention is turned to the biceps tendon insertion. With the forearm in full supination, the radial tuberosity may be approached by using a curved Kelly clamp within the distal biceps tendon sheath, which decreases the risk of violating the ulnar periosteum and causing radioulnar synostosis (Table 1). Once the Kelly clamp is positioned on the far ulnar aspect of the radial tuberosity, the forearm is pronated, and the Kelly clamp is advanced on bone through the interosseous membrane, which can be felt with a pop (Video 1). After this, the Kelly clamp is advanced until palpable on the dorsolateral surface of the forearm, and a 3- to 4-cm longitudinal incision is made over this location. A muscle split through the common extensors is used to approach the radial tuberosity, and this permits full visualization of the biceps tendon footprint. The radial tuberosity is debrided of any remnant soft tissue and biceps tendon stump (Fig 4A). Proper anatomic location can be confirmed under fluoroscopy prior to drilling (Fig 4B). Two 3.2-mm drill pins (Arthrex) are used to drill holes for the cortical buttons, taking care to place these at the proximal and distal aspect of the anatomic biceps insertion to recreate the anatomic footprint (Fig 4C). The Kelly clamp is then used to pass a shuttle suture from distal to proximal to loop around the free limbs of the distal biceps sutures. The shuttle suture is then used to pull the 4 suture limbs and biceps tendon through the interosseous membrane and into the distal incision (Fig 5). Next, the 2 cortical buttons are loaded with their respective pair of sutures, and then the cortical buttons are deployed in the previously drilled holes (Fig 6). With the elbow in approximately 30° of flexion, the 2 suture limbs of each cortical button are sequentially tensioned, until each cortical button firmly abuts the intramedullary cortical surface (Fig 7). One suture from each button is then passed with a free curved needle back through the biceps tendon and then is tied to compress the tendon down to the bone, creating an on-lay effect for tendon-to-bone healing (Video 1). The wound is then copiously irrigated, and a layered closure is performed (Fig 8). Postoperatively, the patient is splinted in approximately 90° of elbow flexion for 2 weeks and then transitioned to a hinged elbow brace, which is worn from 2 to 6 weeks postoperatively. Early range of motion under the supervision of physical therapy begins at 2 weeks, and a strengthening program is started at 10 to 12 weeks postoperatively. Patients are typically fully cleared for all activities at 16 weeks postoperatively.

## Discussion

Multiple surgical techniques exist for treating distal biceps tendon ruptures. This technique employs the Morrey modification of the 2-incision approach,^13^ seeking to optimize anatomic restoration of the footprint while minimizing risks of heterotopic ossification and radioulnar synostosis, which have historically been associated with the 2-incision approach. Our technique is performed through 2 small incisions. The 2-incision approach is associated with decreased rates of injury to the lateral antebrachial cutaneous nerve (Table 2).^15^ Restoration of the 2 heads of the biceps tendon may improve functional outcomes with flexion and supination. Cortical button fixation is associated with lower failure rates and increased load to failure when compared to other fixation methods.^14^^,^^15^ Specifically, unicortical intramedullary button fixation has been validated as a robust fixation method by recent systematic reviews and biomechanical studies.^16^^,^^18^^,^^19^ Another practical advantage includes the surgeon being able to sequentially tension the cortical buttons as appropriate, which avoids potential complications and creates an on-lay effect for effective tendon-to-bone healing. Chronic injuries, revision surgeries, or cases with significant retraction may require a more extensile proximal incision to identify the tendon edges. The use of 2 cortical buttons also raises the cost. The fixation method is easily transferable to a 1-incision technique, if preferred. Overall, this technique facilitates safe and efficient repair of the distal biceps tendon.

## Disclosures

The authors declare the following financial interests/personal relationships which may be considered as potential competing interests: K.A.M. reports a relationship with Arthrex that includes consulting or advisory and speaking and lecture fees. All other authors (E.F., A.R., N.S.) declare that they have no known competing financial interests or personal relationships that could have appeared to influence the work reported in this paper

## Supplementary Data

Video Patient is positioned supine on the operating table. The left upper extremity is draped on a hand table attachment. The camera is positioned above the patient’s left upper extremity viewing the operative field from above.
